# Intrauterine Growth Restriction Is a Direct Consequence of Localized Maternal Uropathogenic *Escherichia coli* Cystitis

**DOI:** 10.1371/journal.pone.0033897

**Published:** 2012-03-21

**Authors:** Michael Bolton, Dennis J. Horvath, Birong Li, Hanna Cortado, David Newsom, Peter White, Santiago Partida-Sanchez, Sheryl S. Justice

**Affiliations:** 1 Section of Infectious Diseases, Department of Pediatrics, Nationwide Children's Hospital, Columbus, Ohio, United States of America; 2 Center for Microbial Pathogenesis, Research Institute at Nationwide Children's Hospital, Columbus, Ohio, United States of America; 3 The Department of Pediatrics and the Center for Microbial Interface Biology, The Ohio State University School of Medicine, Columbus, Ohio, United States of America; 4 The Division of Urology, The Ohio State University School of Medicine, Columbus, Ohio, United States of America; Columbia University, United States of America

## Abstract

Despite the continually increasing rates of adverse perinatal outcomes across the globe, the molecular mechanisms that underlie adverse perinatal outcomes are not completely understood. Clinical studies report that 10% of pregnant women will experience a urinary tract infection (UTI) and there is an association of UTIs with adverse perinatal outcomes. We introduced bacterial cystitis into successfully outbred female mice at gestational day 14 to follow pregnancy outcomes and immunological responses to determine the mechanisms that underlie UTI-mediated adverse outcomes. Outbred fetuses from mothers experiencing localized cystitis displayed intrauterine growth restriction (20–80%) as early as 48 hours post-infection and throughout the remainder of normal gestation. Robust infiltration of cellular innate immune effectors was observed in the uteroplacental tissue following introduction of UTI despite absence of viable bacteria. The magnitude of serum proinflammatory cytokines is elevated in the maternal serum during UTI. This study demonstrates that a localized infection can dramatically impact the immunological status as well as the function of non-infected distal organs and tissues. This model can be used as a platform to determine the mechanism(s) by which proinflammatory changes occur between non-contiguous genitourinary organs

## Introduction

Adverse perinatal outcomes include both prematurity (birth prior to 37 weeks) and/or low birthweight (weight below 2500 grams). There is a discernible etiology in only 50% of adverse perinatal outcomes [1]. The primary known causes of adverse outcomes include multiple gestation [2], high blood pressure [3], diabetes mellitus [4], intrauterine infection [5], pneumonia [6], [7], periodontal disease [8], [9] and UTI [10], [11]. Premature and low birthweight neonates have increased rates of morbidity and mortality in the first year of life and suffer from a plethora of life-long health conditions including: neurological, respiratory, gastrointestinal, cardiovascular, and immunological [12], [13]. These health conditions are more severe when the mother experiences infections during pregnancy [14].

Pregnancy is a unique situation in which the fetus carries both maternal (“self”) and paternal (“non-self”) antigens. While the immune system normally functions to attack non-self stimuli, successful gestation requires the maternal immune response to “ignore” paternal antigens produced by the fetus (termed: fetal tolerance). While mechanisms of tolerance between mother and fetus are not completely understood, it is clear that dendritic cells (DCs), T cells and natural killerd (NK) cells play crucial roles at the maternal-fetal interface and in maternal tissues to ensure that pregnancy proceeds successfully [15]. Immature dendritic cells (iDCs) migrate from the uterus to local lymph nodes during pregnancy to induce T cell differentiation into Th2/3 and regulatory T cells (Treg). Th2/3 cells prevent maternal immune responses from attacking the developing outbred fetus to maintain healthy pregnancies [15].

Maternal infections contribute to nearly 40% of all adverse perinatal outcomes [13]. Murine models have begun to illuminate immunological changes that lead to adverse perinatal outcomes due to maternal intrauterine infection [16], [17], [18]. Various cellular and soluble immune effectors are known to contribute to fetal tolerance and thus, a successful pregnancy. However, these same effectors may negatively affect the developing fetus in the presence of a pro-inflammatory stimulus (e.g. infection). For example, interferon gamma (IFN-γ) and tumor necrosis factor (TNF) promote DC maturation in response to an inflammatory/infectious stimulus [15]. Thus, mature DCs (mDCs) may travel systemically and, in the presence of IL-12, induce T cell differentiation into Th1 type cells [15]. Th1 cells then produce IL-12, IFN-γ and TNF, which result in an inflammatory, unfavorable environment for a developing fetus [15]. Local intrauterine infections may elicit sufficient inflammatory cytokines that recruit inflammatory cells and thus promote an inhospitable intrauterine environment [19], [20]. However, mechanisms by which localized extrauterine infection, such as UTIs, cause low birthweight and/or preterm birth are unknown.

UTIs affect almost 50% of all women (10% of pregnant women) and manifest in a variety of clinical presentations (i.e., asymptomatic bacteriuria, cystitis, and pyelonephritis) [19]. While many UTIs generally have a mild clinical course with few sequelae in the general population, even asymptomatic bacteriuria places the gestating female at risk for low birthweight offspring and preterm birth [20], [21], [22], [23], [24], [25], [26]. These studies suggest that UTIs act as an independent risk factor for preterm delivery and intrauterine growth restriction-low birthweight (IUGR-LBW) [27].

There is a long-standing murine model for UTIs caused by uropathogenic *Escherichia coli* (UPEC) that has been extensively studied to determine the molecular details of UTI pathogenesis (see recent reviews [28], [29]). Observations made with the mouse model have been confirmed in human urine samples and biopsies of patients infected with UPEC [28], [30], [31], [32], [33], which validates this as an appropriate model for human UTI. We hypothesize that a cascade of immunological events occurs in response to a UTI that affects nearby but uninfected organs such as the uteroplacental unit. To this end, we developed a novel murine model for UTI-mediated adverse perinatal outcomes (i.e., IUGR-LBW) and analyzed cellular and soluble immune effectors that contribute to perinatal morbidity.

## Results

### Maternal UTI results in low-birth weight offspring

**Figure 1:**
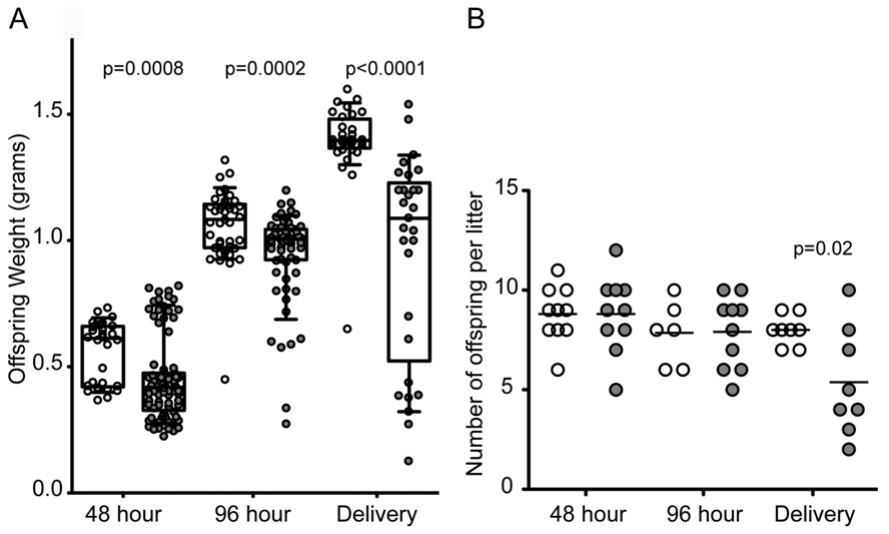
Perinatal outcomes in the presence and absence of maternal UTI. (A) Weight (grams) of each individual offspring from pregnant mice that received sham treatment (white) or experimental UTI (gray) at 48 h (10 mice each cohort), 96 h (10 mice each cohort), and delivery (8 mice each cohort). Box portion of the plot represents 95% of the samples with the range of samples indicated by the external bars; the horizontal bar within the box depicts the median and the mean values are indicated in the text. (B) The number of pups delivered for each mother is depicted. Statistical significance was determined using a two-tailed Mann-Whitney test for internal comparisons of each time point indicated in each panel.

We selected inbred C57Bl/6J females (MHC haplotype b) for mating with inbred CH3/HeN males (MHC haplotype k) to produce outbred fetuses (see Methods and Materials for detailed discussion of the mating). Experimental UTI was established in pregnant mice 14 days after conception, as previously described [34](see Methods and Materials). The genitourinary tissues, fetuses and blood from pregnant mice were harvested at gestational days 16, 18 and after natural parturition (48 hours, 96 hours and 144 hours post infection, respectively). The weight of each offspring was measured on the day of harvest. There was no difference in the viability of the offspring in either experimental cohort either experimental cohort of the fetuses prior to delivery or of the pups observed. Viability was accessed by observation of visible independent movement of each fetus or pup within a given litter. Sham treated mice fostered more robust fetal growth than the mothers that received experimental UTI (Figure 1). At 48 hours post-infection, the median fetal weight from mothers that experienced sham infection was 0.6170±0.122 g, while the median fetal weight from mothers with UTI was significantly lower (0. 417±0.165 g) (p = 0.0008). Fetal weight gain was observed over the next 48 hours in most mothers, but the median remained stunted at 96 h in the experimental UTI cohort (1.093±0.108 g vs 1.004±0.175 g) (p = 0.0002). The greatest disparity in fetal weight was observed at delivery. After natural parturition, the median pup weight from mothers that experienced experimental UTI was 1.090±0.404 g, which was significantly lower than the median pup weight of 1.400±0.08 g from mothers who received sham treatment (p<0.0001). The severity of IUGR is most likely underestimated for the final two days of gestation as mice typically consume pups with weights less than 0.5 g. In fact, the number of pups was significantly different at the time of delivery (Figure 1), although the number of implantation sites was not different (median number of sites, n = 8 for both cohorts, p = 1.0), indicating that the same number of fetuses arose from the conception (data not shown). All mothers gained weight at similar rates and pups were delivered at 20 days (Figure S1). Therefore, the changes in offspring weight cannot be attributed to differences in maternal weight gain or gestational length. Thus, the experimental evidence strongly suggests that IUGR and LBW are the direct consequence of maternal UTI.

### Cystitis remains localized during pregnancy

The genitourinary tissues, fetus and blood from pregnant mice were cultured for viable UPEC to determine the tissue distribution of UPEC following introduction into the urinary tract. No bacteria were detected in any of the tissues analyzed from sham-treated mice. Viable bacteria were present throughout pregnancy and parturition in the bladders of mothers that received UPEC (Table 1). The bacterial burden at the later time points is indicative of a latent infection [35], [36], [37]. Viable bacteria were not detected in the kidneys from mice that were given experimental cystitis, indicating that the urinary tract infection was restricted to the lower tract (bladder) throughout pregnancy. No viable bacteria were detected within the uteroplacental tissue, blood, and fetal tissues (Table 1), indicating that UPEC did not disseminate systemically and that other bacterial species did not gain access to the reproductive organs or the fetuses. Thus, the fetal detriment detailed above is likely not due to the direct bacterial invasion of the uteroplacental tissue observed with other models, but rather is the result of an inflammatory response disseminated to the uterus via cellular and/or soluble immune effectors.

**Table 1:** Bacterial burden of the bladder, kidney, and uterus of infected mothers at 48 h, 96 h, and delivery are reported as colony forming units (CFUs) per individual organ with the standard deviation.

Time	Bladder	Kidney	Uteroplacental	Blood	Fetus
48 hour	6×10^5^+670	N.D.	N.D.	N.D.	N.D.
96 hour	4×10^4^+3887	N.D.	N.D.	N.D.	N.D.
Delivery	1×10^4^+22	N.D.	N.D.	N.D.	N.D.

N.D. = not detected (Limit of detection is 10^3^ cfu).

### Influx of Polymorphonuclear neutrophils (PMNs) and Macrophages in the bladder during UTI

**Figure 2:**
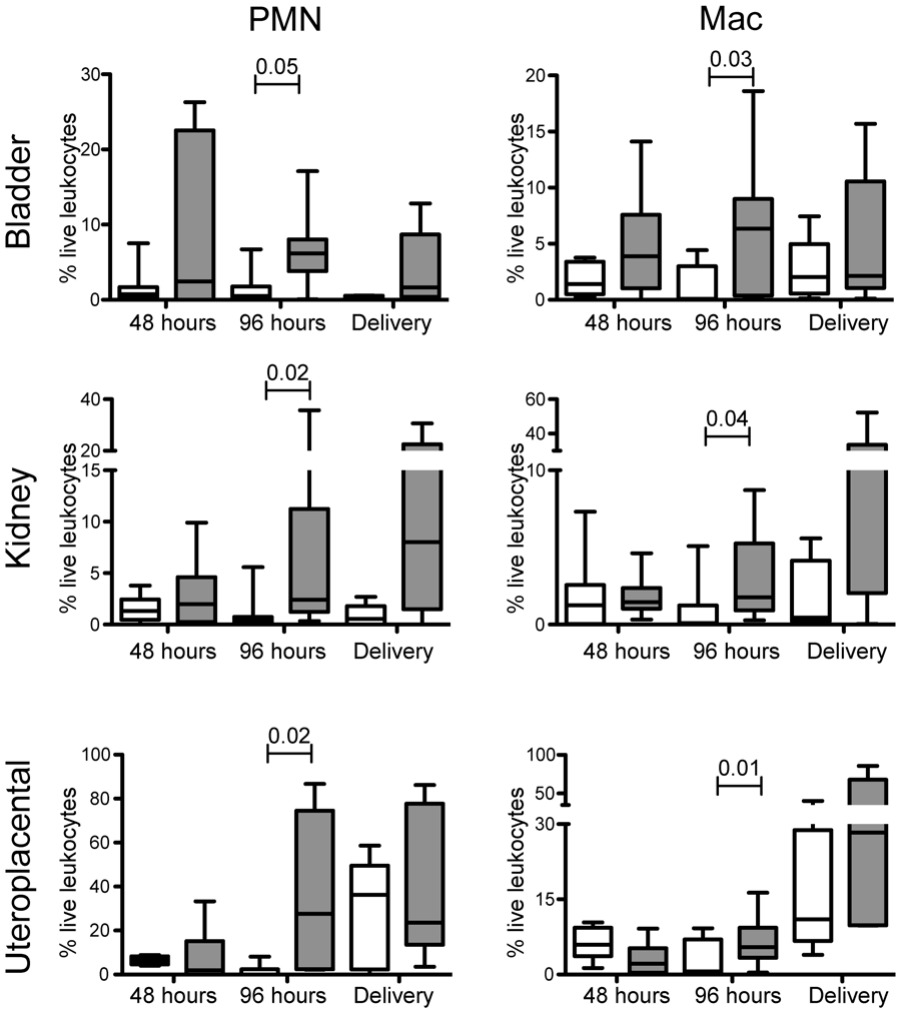
PMN and macrophage infiltration in uteroplacental tissues. The magnitude of the particular cell type of interest is reported as a percentage of live leukocytes within each individual organ indicated from pregnant mice that received sham infection (white) or experimental UTI (gray) at 48 h (10 mice each cohort), 96 h (10 mice each cohort), and delivery (8 mice each cohort). Abbreviations: PMN, polymorphonuclear neutrophil; Mac, macrophage. Bars indicate standard deviation. Statistical significance determined using a two-tailed Mann-Whitney for internal comparisons within each time point indicated in each panel.

Although several laboratories, including ours [38], [39], [40] have described the infiltration of both PMNs and macrophages into the bladder during cystitis, the extent of phagocytic recruitment to the bladder of a pregnant mouse is not well characterized. We next evaluated the magnitude and types of proinflammatory cells recruited to the bladder during experimental UTI in pregnant mice. We initially sought to analyze the bacterial burden, myeloid infiltrate and lymphoid infiltrate in each of the genitourinary tissues. Under our experimental conditions, the samples were divided for three separate quantitative analyses (bacterial burden, myeloid cells, lymphoid cells). We successfully enumerated bacteria (Table 1) and myeloid cells (Figure 2). However, we did not detect a significant magnitude of lymphoid cell recruitment within the portions of the tissue examined at the selected time points (data not shown). At 48 hours post-infection (gestational day 16), both PMNs (CD11b^+^, Gr-1^+hi^, Ly6C^+^, F4/80^−^) and macrophages (CD11b^+^,Gr-1^+low^, Ly6C^+^ F4/80^+^) (Figure S2) were elevated in the bladder of infected pregnant mice compared to sham-treated pregnant mice (Figure 2). At 96 hours post-infection (gestational day 18), the number of phagocytic cells increased significantly [PMNs (p = 0.05) and macrophages (p = 0.03)] in the bladder. After the mothers went through natural parturition, the magnitude of both PMNs and macrophages diminished. The magnitude of inflammation remained constant despite a decrease in the bacterial burden at the later time points. The magnitude of PMN infiltrate is greater in the bladder of pregnant mice than in non-pregnant mice [40], further suggesting that pregnancy produces a distinct inflammatory environment in response to an extrauterine infection (i.e., UTI).

### Influx of PMNs and macrophages in other genitourinary tissues during UTI

We next evaluated whether the induction of localized cystitis in the bladder would elicit a cellular immune response in adjacent genitourinary tissues. Renal tissue demonstrates a similar cellular pro-inflammatory infiltrate as observed in the bladder (Figure 2), despite the lack of viable UPEC in the organ (Table 1). The magnitude of both PMNs and macrophages increased over time and reached significance on gestational day 18 (96 hours post-UTI) (p = 0.02 and p = 0.04, respectively). With respect to the uterus, we observed a statistically significant change in infiltration of PMNs and macrophages throughout the last week of gestation (Figure 2; note change in scale of y axis). The magnitude of infiltration of PMNs into the uteroplacental tissue was inversely correlated with fetal weight at 96 h post infection (Figure S3). On gestational day 18 (96 hours post-UTI) the magnitude of PMNs and macrophages was significantly greater in the cohort that received experimental UTI when compared to the sham cohort (p = 0.02 and 0.01 for PMNs and macrophages, respectively). On the morning following natural delivery (gestational day 20), the difference in PMN and macrophage infiltration into the uterus lessened due to increased infiltration in the sham treated group (Figure 2). Phagocytic influx into uterus at parturition has been previously demonstrated [41]. Sukhikh *et al.* suggested a functional role for phagocytes during and after the natural course of parturition. We observed a difference in the magnitude of PMN and macrophage infiltration between the uteroplacental tissue and the non-pregnant uterus (data not shown), suggesting that pregnancy presents a unique situation, which may promote proinflammatory immune responses in the presence of infection. Interestingly, our results indicate that inflammation-associated cellular effectors are present in tissues that lack UPEC colonization, (e.g. uteroplacental tissue) suggesting that either soluble bacterial factors or transient host-derived mediators result in such an inflammatory environment.

### Mature dendritic cells (mDC) increase during UTI

**Figure 3:**
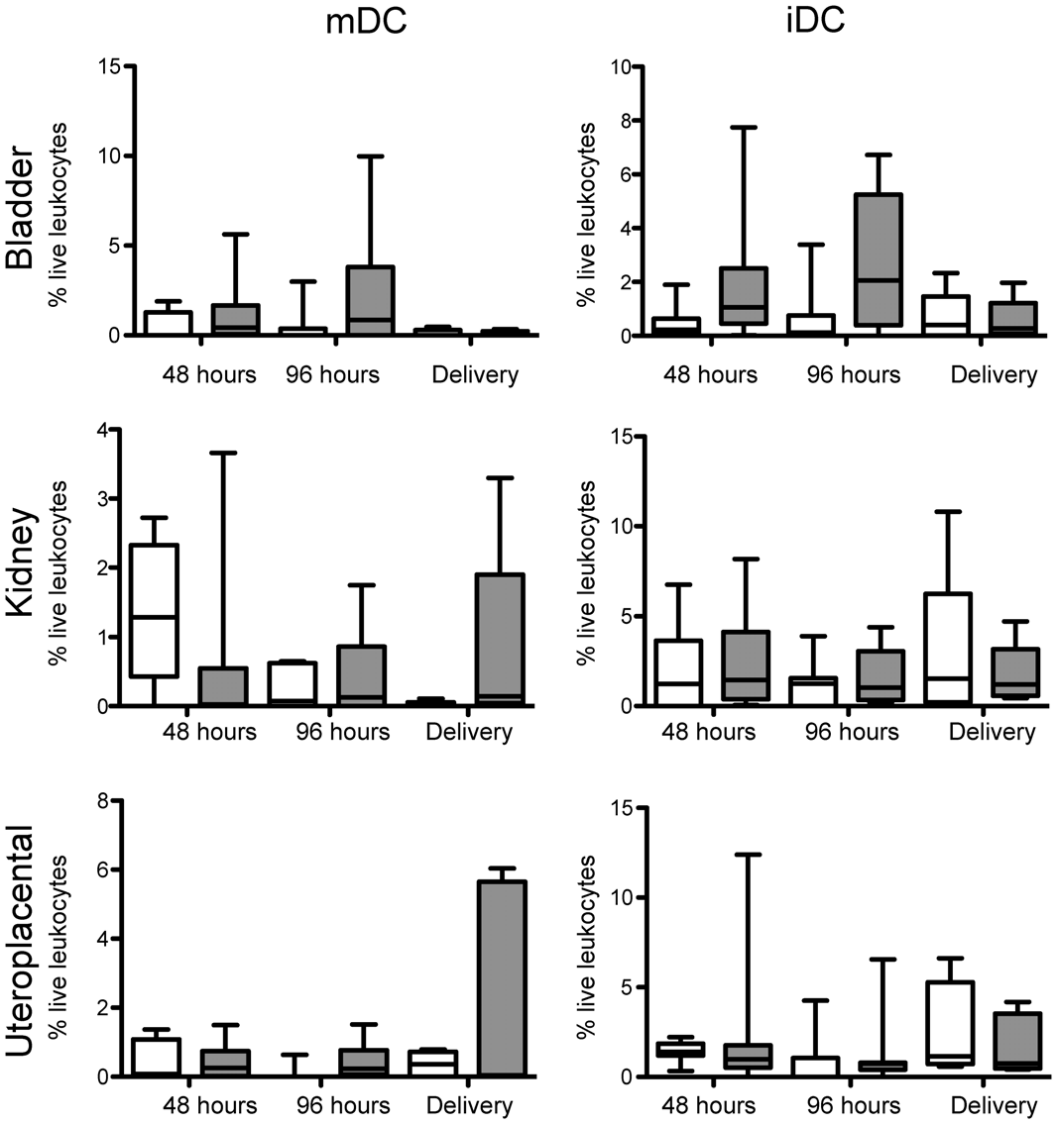
Presence of dendritic cells in uteroplacental tissues. The magnitude of the particular cell type of interest is reported as a percentage of live leukocytes within each individual organ indicated from pregnant mice that received sham infection (white) or experimental UTI (gray) at 48 h (10 mice each cohort), 96 h (10 mice each cohort), and delivery (8 mice each cohort. Statistical significance determined using a two-tailed Mann Whitney for internal comparisons within each time point, no significance observed. Abbreviations: mDC, mature dentritic cell; iDC, immature dendritic cell.

The maturation state of DCs dictates the success or failure of outbred pregnancies [42]. In this study, we detected a statistically significant increase in the magnitude of mDCs (CD11c^+^, MHC II^+med-hi^) in the bladders of infected mice 48 and 96 hours post-infection (gestational day 16 and 18, respectively) (Figure 3). The presence of mDCs in the uterus indicates that inflammatory signals have expanded beyond the bladder and initiation of adaptive immunity has occurred (i.e., uterine mDCs). Interestingly, the percentage of iDCs, (CD11c^+^, MHC II^+lo^) which are important for maintenance of fetal tolerance, remained unchanged in uteroplacental tissues at all of the post-infection time points analyzed (Figure 3). The similar levels of iDCs suggest that, at least, portions of fetal tolerance are intact in the presence of maternal UTI. In addition, our data suggest that mDCs may have matured elsewhere (e.g., bladder) and may have trafficked to the uterus upon infection of the urinary.

### Serum cytokines are elevated during maternal UTI

**Figure 4:**
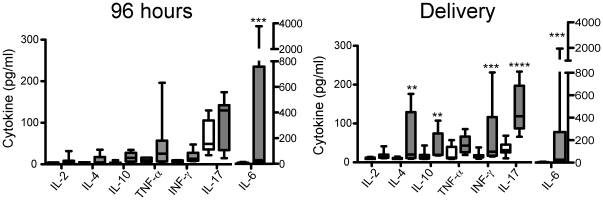
Serum cytokine profiles during pregnancy and UTI. The magnitude of serum cytokines (pg/ml) of cytokines at 96 h (A) and delivery (B) of mothers that received sham infection (white) or experimental UTI (gray). The serum taken from each individual mouse is measured separately. The number of mice used in each cohort at 96 h and delivery were 10 and 8, respectively. Statistical significance was determined using two-tailed Mann-Whitney (**, p<0.04; ***, p<0.008; ****, p = 0.0003).

As serum levels of pro-inflammatory cytokines are elevated during UTI [33], we were interested to determine whether specific cytokines are associated with maternal UTI-mediated IUGR-LBW. Serum was collected from sham-treated and experimental UTI cohorts at the time of tissue harvest for evaluation of pro-inflammatory cytokine levels as described in Materials and Methods. While cytokines were elevated in the experimental UTI cohort as compared with the sham cohort at 48 hours, none of the individual comparisons reached statistical significance (data not shown). Circulatory pro-inflammatory cytokines were greater in mothers experiencing experimental UTI than in those mothers in the sham cohort at 96 hours post-introduction of UTI and at parturition (Figure 4). At 96 hours post introduction of infection, only the values for IL-6 were significantly greater in mothers that received experimental UTI compared to mothers in the sham cohort (p = 0.0028) (Fig. 4). When the magnitude of serum IL-6 was correlated to the weight of each individual fetus at 96 h post infection, the highest levels of IL-6 were observed in those infected mothers with the lowest weight offspring (Figure S4). At delivery, the magnitude of IL-6 remained significantly elevated (p = 0.001) as well as the magnitude of IL-4 (p = 0.04), IL-10 (p = 0.03), INF-γ (p = 0.008) and IL-17 (p = 0.0003) (Figure 4). These data demonstrate that a robust systemic immunological response, as measured by the magnitude of serum cytokines, occurs downstream of maternal UTI at a time when the bacteria have transitioned from the acute to the latent infection [35], [36], [43].

### Transcriptional changes in placenta resulting from maternal UTI

In order to identify early changes in the placenta that lead to fetal demise, we compared transcriptional changes in the presence and absence of experimental UTI. Placenta and uterus were harvested on gestational day 16 (48 hours post UTI) from mice from the sham cohort and mice from that received experimental UTI on gestational day 14. The tissue samples were processed for extraction of mRNA and subjected to Agilent microarray analysis (Methods and Materials). There were no significant differences in the transcriptional profiles between the uterus tissue in the presence or absence of infection (data not shown). Within the placental tissues, there were 257 transcripts that ranged in differential expression values ranging from 4.7–810 fold (Table 2)(full raw data deposited into the MIAME compliant GEO database, accession GSE32028). Cathepesin Q, as well as a number of other apoptotic factors, demonstrated robust increase in expression (up to 810 fold) in the presence of maternal UTI. Placental levels of prolactin were also significantly increased in the presence of maternal UTI. There was an increase in the expression of the leptin receptor in the placenta of mothers that receive experimental UTIs. None of the genes represented in the transcriptional analysis were directly related to activation or recruitment of the innate immune response (e.g. TRL receptor, cytokine production, chemokine production), which corroborates the observation that the reproductive organ is sterile. Furthermore, the lack of primary inflammatory mediators suggests that bacterial antigens do not appear to escape the bladder to gain access to the reproductive organ to initiate the proinflammatory responses observed in the uteroplacental unit.

**Table 2:** The top 4 families that demonstrate increased transcription in each individual placenta at 48 hours following introduction of maternal UTI are indicated, along with the number of times each family was represented and the fold change in expression.

Family	Function	Occurrences	Fold Increase
Cathepsin	Apoptosis	6	81–810
Prolactin	Maintain fetal tolerance	22	28–784
Leptin receptor	Fetal weight gain	9	71–93
Ceacam	Cell adhesion	5	56–568

The full list of transcripts is deposited in the GEO database (accession number GSE32028).

## Discussion

Here, we demonstrate that a non-disseminated UTI is associated with adverse perinatal outcomes. Kaul *et al.* presented a mouse model of pyelonephritis-induced preterm birth and low birth weights while investigating the virulence of the Dr adhesion [44]. In contrast to our model, the mothers became septic and all of the fetuses became infected, presumably due to the use of a pyelonephritic UPEC strain (compared to our cystitis strain) and the use of immunocompromised TLR4-deficient mothers. Due to the dissemination to the reproductive organ, the model presented by Kaul *et al*. provides additional insight into the effects of intrauterine infection on fetal development. In our system, the offspring of mothers that experienced experimental UTI displayed up to 80% decrease in fetal weight when compared to non-infected mothers. This phenomenon occurs as early as 48 hours post introduction of cystitis and continues throughout gestation. An important feature of our model of adverse perinatal outcome model is that localized cystitis induced delayed infiltration of PMNs and macrophages in distal organs. We demonstrated that bladders from mice with experimental UTI had an expected increase of professional phagocytes as a result of localized infection; however, inflammatory cells were also observed in the kidney, even in the absence of viable bacteria. Furthermore, a robust cellular inflammatory response was observed in the uteroplacental tissue of those mice with experimental UTI when no viable bacteria were recovered (the same tissue sample tested for both bacteria and cellular infiltrate). In fact, there was a correlation between PMN infiltration and diminished fetal weight gain (Figure S3), suggesting that the presence of the PMNs may contribute to the IUGR-LBW observed in the presence of non-disseminated UTI. The cellular inflammatory response in the pregnant reproductive organ was more severe than the kidney and in the naïve uterus, suggesting that the uteroplacental tissue is no longer privileged and may be more susceptible than other adjacent genitourinary organs to systemic changes in immune status.

Our evidence indicates that the placenta is progressing through apoptotic death as a consequence of a strong cellular influx of immune cells into the reproductive organ. Cathepsin Q is a placental specific apoptotic factor that induces necrotic cell death in the presence of reactive oxygen species-mediated DNA damage such as produced by PMNs and macrophages. We hypothesize that the induction of Cathepsin Q in the UTI cohort indicates that the influx of professional phagocytes is inducing DNA damage within the placental cells. High serum prolactin is associated with miscarriage in humans [45] and prolactin is involved in the T-cell functions associated with maintenance of fetal tolerance [46]. Whether prolactin levels are elevated as a consequence of the phagocyte infiltration as a means to protect the fetus from the maternal immune response or whether the levels disrupt the T-cell functions that lead to inflammatory cell infiltrate are under investigation. Leptins are important in proper fetal weight gain [47]. The increased production of the leptin receptor suggests that the placental unit is attempting to acquire more nutrition for the developing fetus. These observations provide insight into the molecular mechanisms that underlie intrauterine growth restriction as a result of maternal UTI.

Cytokines measured in the serum of our infected pregnant females demonstrated the absence of Th2 effectors characteristic for healthy pregnancy, and thus, both Th1 cytokines (INF-γ, TNF-α) and Th17 cytokines (IL-17) are significantly elevated at delivery. Furthermore, IL-6 and TGF-β are key factors in the development of Th17 cells. Similar systemic inflammation along with an imbalance of Th1 and Th2 cells in the uterus is a dominant component implicated in the pathogenesis of pre-eclampsia [48]. This change from Th2 to Th1 should alter DC function and compromise fetal tolerance. However, we did not detect significant changes in uteroplacental iDCs, thus, we favor the hypothesis that mDCs may traffic to adjacent organs via hematogenous route. Multiple mechanisms by which DCs can mobilize to lymph node or spleen, return to the blood and enter other organs were reviewed by Randolph *et al*. [49]. Reverse trafficking might facilitate the spread of antigens from tissue to tissue carried by DCs serving as Trojan horses [50], [51]. However, our transcriptional analysis of the placenta indicates that the placenta is not the primary site of immune activation under these experimental conditions. It is also possible that cellular innate responses result in increased levels of cytokines such as TNF-α that could stimulate DC maturation in distinct and distant urogenital tissues, which would in turn, promote Th1/Th17 responses and manifest in a more robust systemic inflammation. Indeed, we were able to detect an increase in mDC in the uteroplacental tissue, and also elevated inflammatory cytokines in the serum, which might impact the type and severity of the inflammatory responses. The implication of a remote infection producing sufficient inflammatory stimuli to restrict adequate fetal development has numerous clinical implications. Moreover, these observations suggest that local infections result in the inflammation in a number of non-infected organs, which may also impact the function of those organs. In this particular case, the systemic inflammation has a dramatic effect on an organ function (i.e. the uteroplacental unit) leading to intrauterine growth restriction and low birth weight.

In conclusion, our murine model of maternal non-disseminated UTI-induced adverse perinatal outcomes provides a platform to further elucidate the inter-organ cross talk that occurs during localized infections.

## Materials and Methods

### Mice and optimized mating for generation of outbred pregnancies

4–6 week old C57Bl/6 female mice were obtained from Jackson Lab, (Bar Harbor, ME). Females were outbred with C3H/HeN males less than 1 year of age purchased from Harlan Labs (Indianapolis, IN). Forty-eight hours prior to the overnight mating sessions, males and females were exchanged in the cages to allow the mice to become familiar with their future mate's scent in an attempt to increase the rate of conception. To synchronize impregnation and maintain precise infection and delivery dates, 2 females were housed with 1 male (in the female's cage) overnight for less than 16 hours. Therefore, successful pregnancies were determined by maternal weight gain following cohousing. Female mice that did not become pregnant as determined by every other day weight measurements were returned to the mating rotation every 2–3 weeks until conception occurred. Females were deemed pregnant if they had gained at least 2 grams over a 1-week period. The distinct histocompatibility complex classes chosen will invoke fetal tolerance to prevent fetal rejection. Our mating combination resulted in the typical number of fetuses with a gestational length of 20 days (Figure S1). With this mating pair combination, we observed a normal distribution of fetus number (8–10) and gestational length (typically 19–21 days). Taken together with an average rate of impregnation of 30%, these variables indicate that maternal fetal tolerance prevented rejection of the paternal allo-antigens resulting in a successful pregnancy. The presence of fetal tolerance in our outbred model is further supported by other investigations that demonstrate fetal rejection using a different outbred mating pair that does not invoke fetal tolerance [52], [53]. Maintenance of all mice was in strict accordance of the Institutional Animal Care and Use Committee (IACUC) rules and regulations. All animals are housed in accordance with USDA guidelines for the care and housing of laboratory animals, and USDA officials routinely inspect the facilities. Specifically, the mice had a normal 12-hour light-dark cycle and were maintained on standard chow diet (Harlan Laboratories). The mice were housed using ventilated cages, corncob bedding, and proper enrichment with changes on a two-week basis. As such, at most, the cages were changed once during the experimental period. The mice were also sequestered from being inadvertently disturbed by human interactions due to housing in a separate containment unit that was only opened when the pregnant mice were monitored. The experiments presented in this manuscript are approved (AR08-00039) by The Research Institute at Nationwide Children's Hospital Institutional Laboratory Animal Care and Use Committee (Welfare Assurance Number A3544-01).

### Infection/Bacterial Strains

UTI89/pANT4 [43], [54], a prototypical UPEC strain obtained from a patient with cystitis [35] which contains an episomal plasmid asserting ampicillin resistance, was used for all studies. Pregnant female mice were transurethrally inoculated with 50 µl of ∼10^8^/mL UTI89/pANT4 or sterile phosphate buffered saline (PBS) as previously described [34].

### Tissue Processing

Following isoflurane anesthesia per IACUC protocols, renal arteries of the pregnant mice were severed and blood was aspirated into tubes containing EDTA. Bladder, kidneys, and uteroplacental tissues were minced into small pieces with scissors and were digested for 25–30 minutes at 37°C under slight agitation. Bladders and kidneys were digested in 0.5 mg/ml collagenase and 100 µg/ml DNase I in RPMI 1640 medium (Invitrogen, Grand Island, NY) containing 0.5% heat-inactivated fetal calf serum (PAA Laboratories, Pasching, Austria) and 20 mM HEPES as previously described [55], [56]. The uteroplacental tissues were digested in a HBSS solution containing 1 mg/ml collagenase type IV, 0.2 mg/ml DNase I, 200 U/ml hyaluronidase, and 1 mg/ml bovine serum albumin/fraction V as previously described [57]. After enzymatic dissociation of tissues, an aliquot of each organ (bladder, kidney, or uterus) or undiluted blood was serially diluted in PBS and plated on LB agar and LB agar containing ampicillin to enumerate bacterial burden. The number of colonies was indistinguishable on LB agar and LB agar containing ampicillin.

### Analysis of Cellular inflammation

Cell suspensions were washed in FACS buffer and filtered through a 70 µm nylon filter (Becton Dickinson, Franklin Lakes, NJ) to remove cellular debris. One mL of ACK lysing buffer (Invitrogen, Grand Island, NY) was added to the kidney, spleen, and bone marrow to lyse red blood cells. A discontinuous 20, 40, and 80% Percoll gradient was prepared as described [58] according to manufacturers recommendations (GE Healthcare Biosciences, Pittsburgh, PA). One ml of single cell suspensions of kidneys and uterus were underlayed in 80% Percoll layer and centrifuged at 500× g for 25 min at room temperature. The band between the 40 and 80% layer was collected and washed twice in PBS. Prior to antibody staining, single cell suspensions of each sample were incubated with Fc blocking antibody (eBiosciences, San Diego, CA), to minimize non-specific antibody staining. The following antibodies were used to discern specific cellular populations: anti-CD11b-phycoerythrin (M1/70), anti-Gr-1-phycoerythrin-cy-5.5 (RB6-8C5), anti-Ly6C-Alexaflour 488 (ER-MP20), anti-CD11c-allophycocyanin (N418), anti-major histocompatibility complex class II-Alexa 700 (M5/114.15.2), anti-major histocompatibility complex class II-Biotin (KH74), anti-allophycocyanin-cy-7 streptavidin, anti-F4-80-phycoerythrin-cy-7 (BM8) (all from eBiosciences, San Diego, CA except anti-MHC II –Biotin, BD Pharmingen, San Diego, CA). Single color controls for each antibody were used with either bone marrow or spleen for fluorescence compensation. A viability discrimination marker was used according to the manufacturers' instructions (Violet Live/Dead kit, Invitrogen, Carlsbad, CA) to exclude dead cells from subsequent analysis. Cellular data were collected with a BD LSR II flow cytometer (Becton Dickinson, San Jose, CA) and analyzed with Flowjo software (Tree Star, Ashland, OR). Statistical analysis and graph constructions were made using Prism 5.0 (GraphPad Software, Inc, La Jolla, CA).

### Bacterial Cultures

Each organ sample, including blood, was plated on LB plates containing ampicillin sulfate (125 µg/ml, Fischer Scientific, Fairlawn, NJ). Serial dilutions of each organ were plated in consecutive rows on LB agar and LB agar containing ampicillin to enumerate bacterial burden. Undiluted blood samples were also plated. Colony counts were then recorded for any growth and labeled as none detected if no growth was witnessed. The number of colonies was indistinguishable on LB agar and LB agar containing ampicillin.

### Cytokine Analysis

Maternal serum was analyzed for various cytokine levels using a Th1/Th2 and a Mouse Inflammation Cytokines Bead Arrays (both from BD Biosciences, San Jose, CA). Samples taken at the time of harvest from severed renal vessels were spun for 10 minutes at 10,000 rpm. Serum was then collected and frozen at −80°C for batch testing. Fifty microliters of serum was used for each sample tested. If more than 50 µl was available, samples were run in duplicate. We then used an LSR II cytometer to measure the following: IL-2, IL-4, IL-5, IL-6, IL-10, IL-12, IL-17, IFN-γ, and TNF-α. Software included in the kits was used for conversion into pg/ml.

### mRNA Extraction

Samples of the placenta and the uterus were taken on gestational day 16 from 4 mice. Two mice received experimental UTI or shame inoculation on gestational day 14. Tissue samples were homogenized in 1 ml TRIZOL® Reagent per 50–100 mg of tissue using power homogenizer Polytron. The homogenized samples were incubated for 5 minutes at room temperature and then 0.2 ml of chloroform per 1 ml of Trizol were added to the tube. Total RNA was isolated from the homogenized samples with TRIZOL reagent according to the manufacturer's recommendations.

### Microarray analysis

Two-color gene expression analysis was performed in-house by the Biomedical Genomics Core. In outline, 500 ng of total RNA was amplified and labeled with either Cy3 (uninfected control samples, n = 2) or Cy5 (infected test sample, n = 2) using the Low Linear Amplification Kit (Agilent Technologies, CA). This labeling reaction produced 7–10.0 µg of Cy3-labeled cRNA (anti-sense), by first converting mRNA primed with an oligo (d)T-T7 primer into dsDNA with MMLV-RT and then amplifying the sample using T7 RNA Polymerase in the presence of Cy3-CTP. After purification, 825 ng of each the test and control cRNA was fragmented and co-hybridized to the Whole Mouse Genome Oligo Microarray (AMADID 04868; Agilent Technologies, CA) array for 17 hr. at 65°C. This array consists of 44,000 60-mer oligonucleotides, representing 21,609 known genes represented by 33,661 transcripts.

Microarray slides were washed and then scanned with an Agilent G2505C Microarray Scanner. Images were analyzed with Feature Extraction 10.7 (Agilent Technologies, CA) in two color gene expression mode. Median foreground intensities were obtained for each spot and imported into the mathematical software package “R”. The intensities were corrected for the scanner offset but not further background corrected. The dataset was filtered to remove positive control elements. Using the negative controls on the arrays, the background threshold was determined and all values less than this value were set to the threshold value. Finally, the data were global loess normalized using the LIMMA microarray processing package in “R” (Smyth, 2004). Fold change values were then calculated for each element on the array and averaged for the replicate samples. Genes with a fold change >2 fold up or down were considered differentially expressed. All microarray data is MIAME compliant and the raw transcriptional data has been deposited into the MIAME compliant database, GEO, accession number GSE32028.

### Statistical Analysis:

Exact values (symbols) or median values (graphs) are represented in each figure. Statistical significance was determined using two-tailed non-parametric Mann-Whitney U test (GraphPad Software, Inc, La Jolla, CA).

## Supporting Information

Figure S1 **Maternal weight gain during UTI.** Mothers were weighed daily following impregnation to validate pregnancy. On gestational day 14, mothers received PBS (black lines) or experimental UTI (gray lines) and were weighed at least every other day to assess the effect of UTI on maternal weight gain. The rate of gain (slope of the line) are not different between the cohorts. Only three representative mothers from the sham cohort and 7 of the UTI cohort are depicted for clarity.
(TIF)

Figure S2 **Gating strategy for identification of infiltrating myeloid cells.** Representative graphs indicate the markers and the populations that were selected for analysis.
(TIF)

Figure S3 **Correlation of PMN infiltration with IUGR.** The magnitude of PMN infiltration into the uteroplacental tissues was inversely correlated with the weight of each offspring at 96 hours in pregnant mice that received experimental UTI (gray) or sham infection (black). Each data point represents the weight of a single fetus with the magnitude of the PMN infiltration into the uteroplacental tissues.
(TIF)

Figure S4 **Correlation of IL-6 with IUGR.** The magnitude of IL-6 serum levels was inversely correlated with the weight of each offspring at 96 hours in pregnant mice that received experimental UTI (gray) or sham infection (black). Each data point represents the weight of a single fetus with the magnitude of the maternal circulatory IL-levels.
(TIF)
