# Supplementary figures and images for: Intrauterine Growth Restriction Is a Direct Consequence of Localized Maternal Uropathogenic Escherichia coli Cystitis
Source: PLoS One. 2012 Mar 21;7(3):e33897. doi: 10.1371/journal.pone.0033897 (PMC3309957; doi:10.1371/journal.pone.0033897)

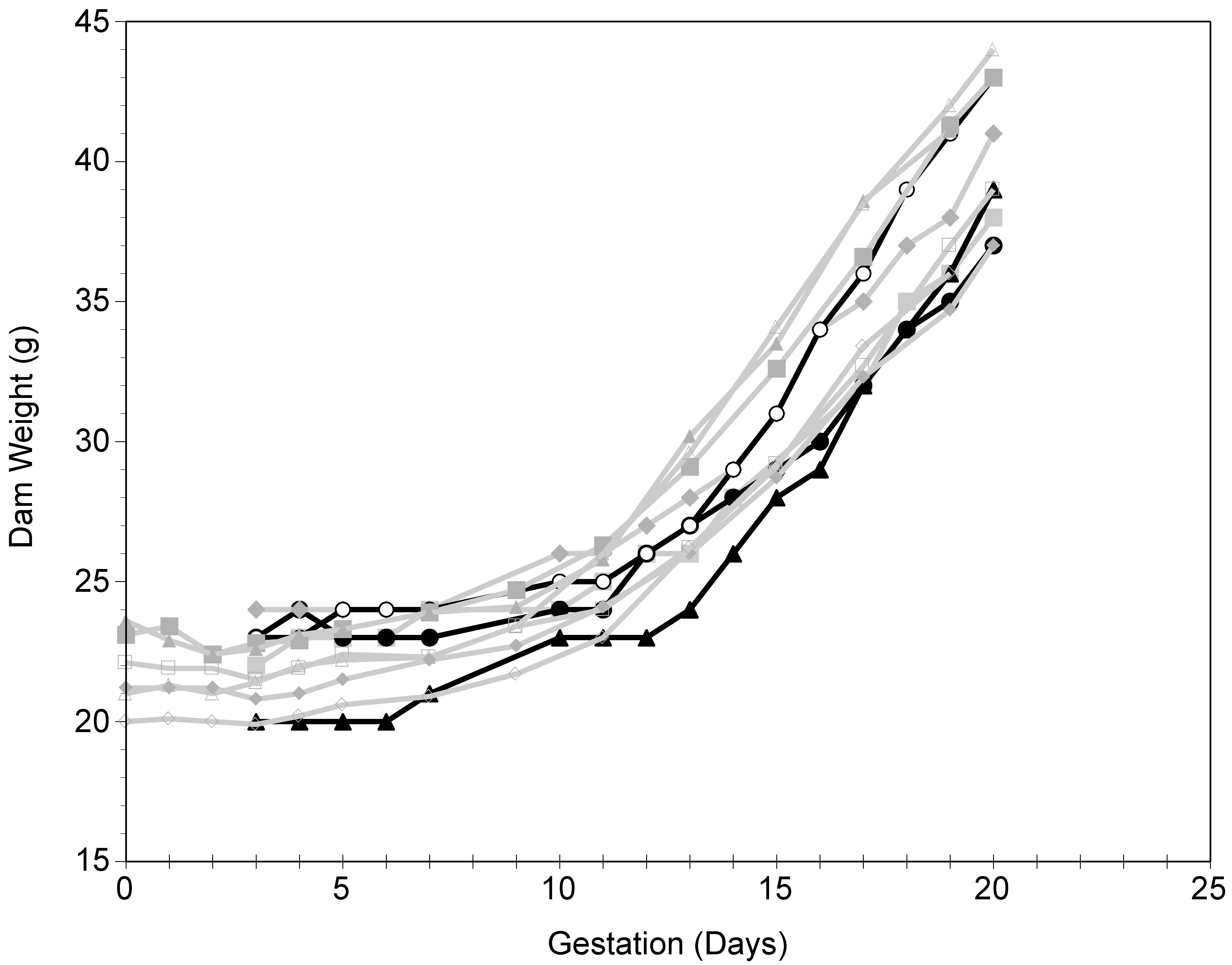

Supplement: Figure S1 — Maternal weight gain during UTI. Mothers were weighed daily following impregnation to validate pregnancy. On gestational day 14, mothers received PBS (black lines) or experimental UTI (gray lines) and were weighed at least every other day to assess the effect of UTI on maternal weight gain. The rate of gain (slope of the line) are not different between the cohorts. Only three representative mothers from the sham cohort and 7 of the UTI cohort are depicted for clarity. (TIF) [file pone.0033897.s001.tif]

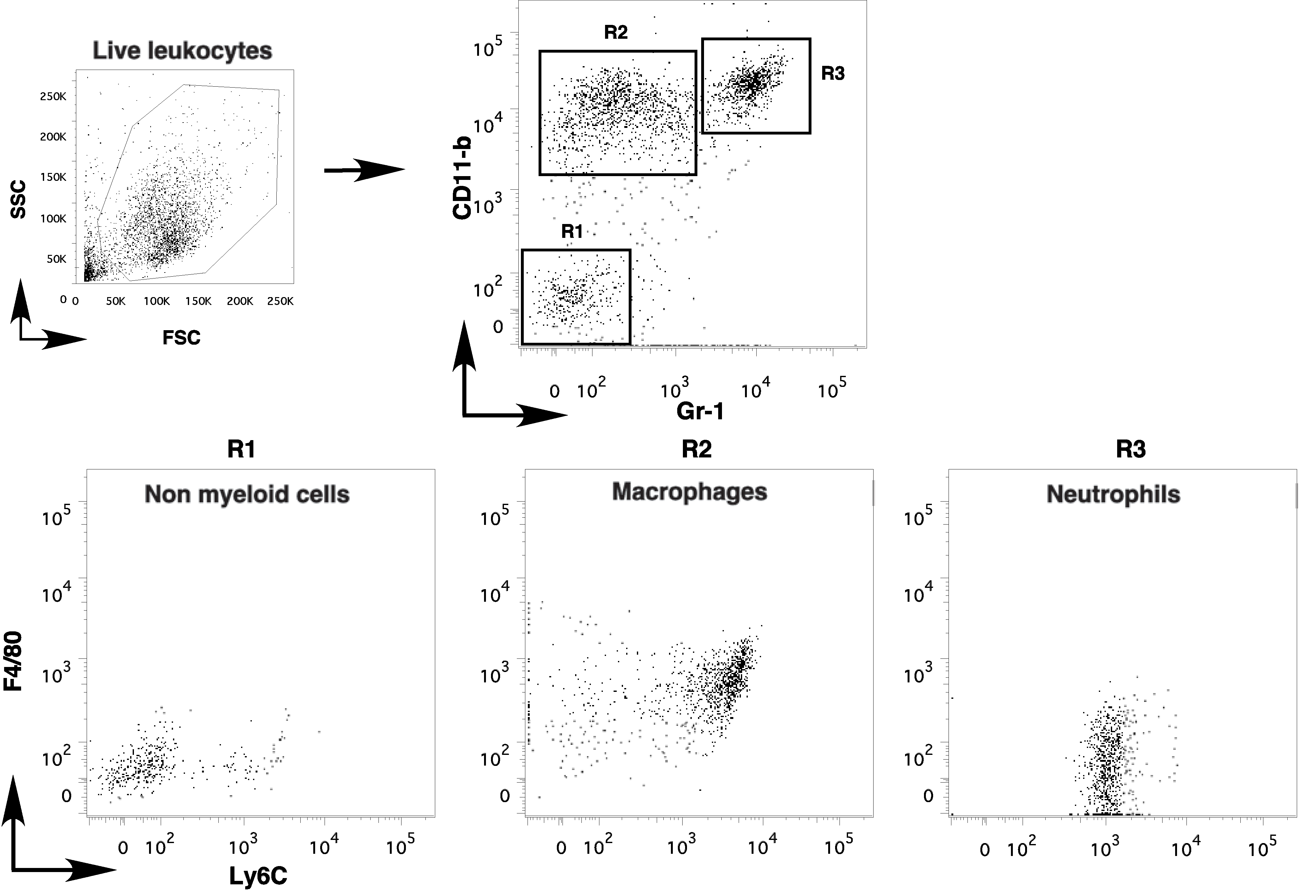

Supplement: Figure S2 — Gating strategy for identification of infiltrating myeloid cells. Representative graphs indicate the markers and the populations that were selected for analysis. (TIF) [file pone.0033897.s002.tif]

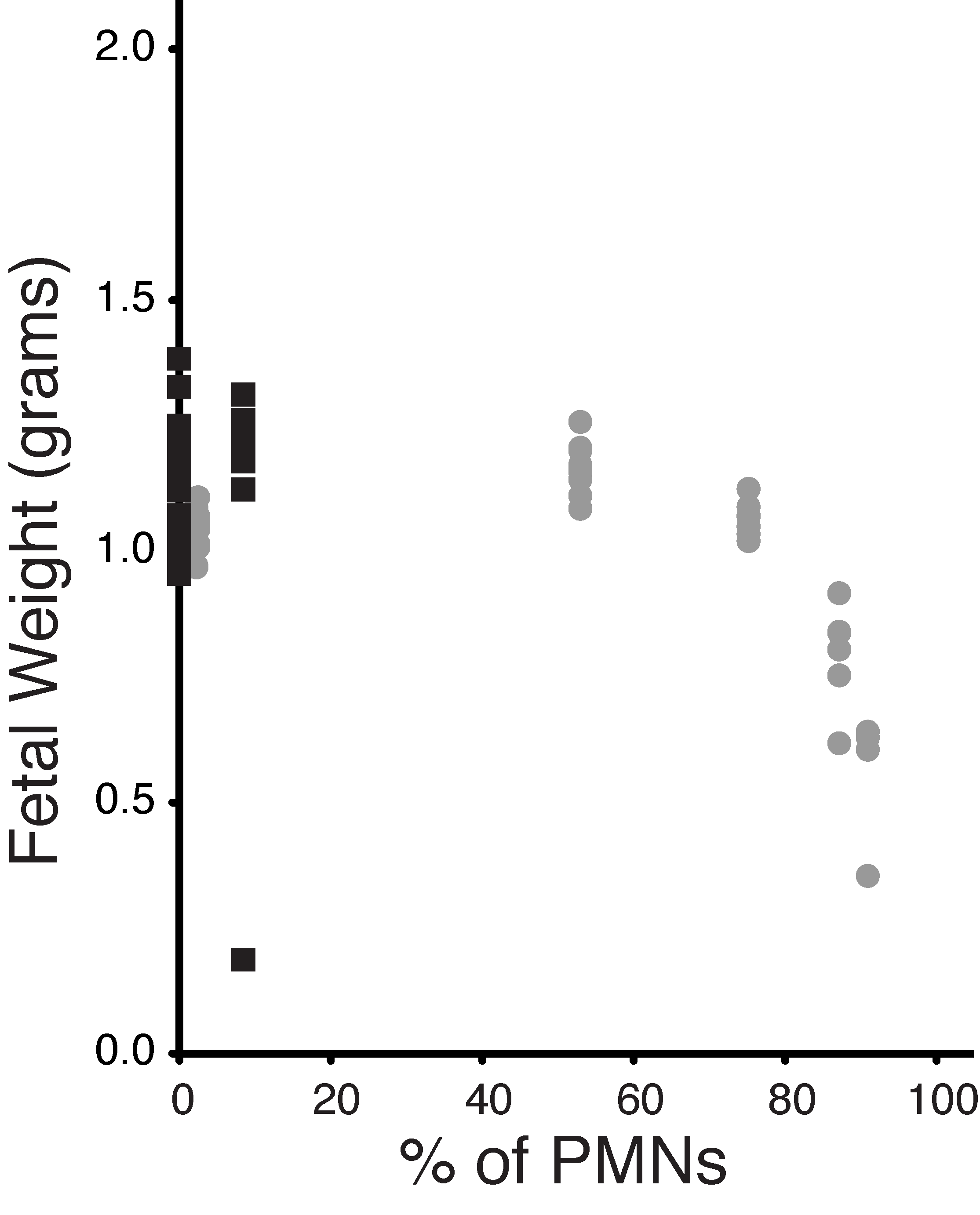

Supplement: Figure S3 — Correlation of PMN infiltration with IUGR. The magnitude of PMN infiltration into the uteroplacental tissues was inversely correlated with the weight of each offspring at 96 hours in pregnant mice that received experimental UTI (gray) or sham infection (black). Each data point represents the weight of a single fetus with the magnitude of the PMN infiltration into the uteroplacental tissues. (TIF) [file pone.0033897.s003.tif]

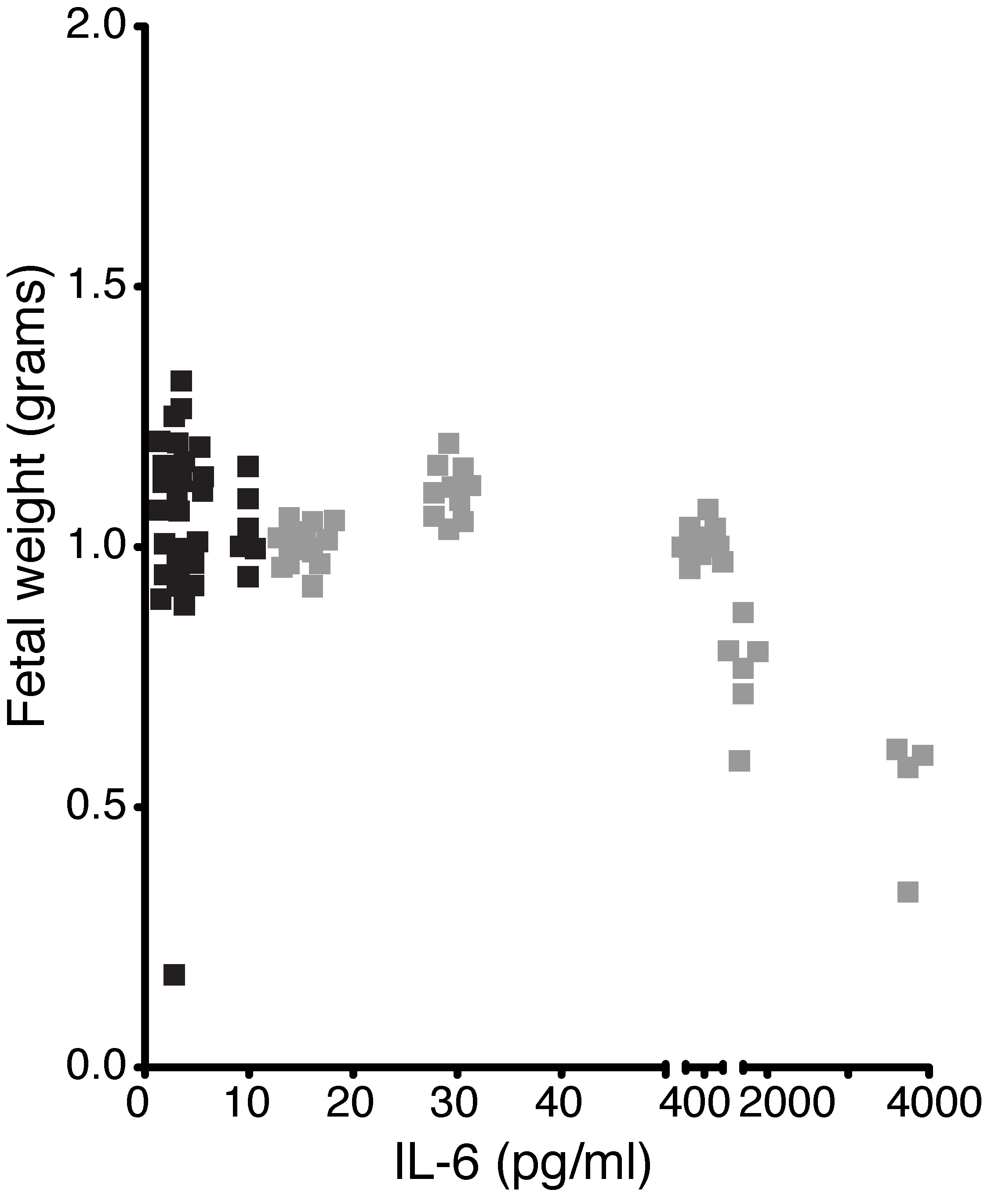

Supplement: Figure S4 — Correlation of IL-6 with IUGR. The magnitude of IL-6 serum levels was inversely correlated with the weight of each offspring at 96 hours in pregnant mice that received experimental UTI (gray) or sham infection (black). Each data point represents the weight of a single fetus with the magnitude of the maternal circulatory IL-levels. (TIF) [file pone.0033897.s004.tif]
